# Hydrobromic Acid in Cerebro Spinal Meningitis

**Published:** 1878-05-20

**Authors:** 


### HYDROBROMIC ACID IN CEREBRO SPINAL MENINGITIS.

The many excellent qualities of this acid which render it so useful a member of therapeutical armamentarium, especially in fevers accompanied with considerable disturbance, makes it incumbent upon us to refer to it briefly in this issue. There has been considerable desultory writing in the journals concerning it during the past few months, all pointing to its excellent qualities as a cerebral sedative, and tranquilizer of the nervous system. It possesses all the beneficial action of the bromide of potassium, without the relaxing effects of the potash, and does not superinduce boils. It does not stimulate as does bromide of ammonium, and may be readily combined with quinine, to produce the hydrobromate of quinine, a most valuable tonic to the nervous system in low forms of fever, etc.

To Dr. Fothergill of London, England, belongs the credit of first having separated this acid for use, since which time it has excited considerable interest in medical circles. He gives the following formula for its production in quantities of two quarts: dissolve dr. xj of bromide of potassium in four pints of water, then add dr. xiij of tartaric acid. A precipitate of bitartrate of potash falls down as a sediment, and the hydrobromic acid remains in a clear, bright, almost colorless fluid, possessing an acid taste and the ordinary acid properties, and is possessed of the peculiar therapeutical properties of bromide of potassium, as distinguished from those of any other salt of potash. The dose of this acid, thus prepared, is from half a drachm to a drachm. The smaller dose is usually that employed, except in severe cases. It is the form of bromine best suited for use in medicine. It is commending itself in the South as a remedy in fever, combined with large anti-pyretic doses of quinine. In the *Peninsular Journal of Medicine*, Dr. Wade recommends its use in the treatment of fevers and says "it would seem the acid *par excellence* when there is much cerebral excitement, in pyretic affections."

In cerebro-spinal meningitis, we have a specific contagious virus of a typhous nature, attacking with especial virulence the great nerve centres. To treat this successfully requires the highest skill, and greatest promptitude and aptitude in the selection of remedies. Briefly, we may here summarize the most recent conclusions of the ablest men in the profession as to its treatment, as in this we may best shew the place and power of this acid, as an agent in the treatment of this formidable affection.

First: the hyperæmia of the brain and spinal eord should be relieved by the prompt and repeated application of leeches, until the pulse has fallen to below 100 or within a point at which it ceases to be alarming. Second: hot applications (not cold) are to be applied to the head and spine, with mustard pedi-luvia. Third: the bowels should be unloaded with an active cathartic. Fourth: to relieve the hyperpyrexia (the temperature being sometimes as high as 104° or even 106°) sedative doses of quinine (say 2 to 5 or even 10 grs.) with oz. j doses of the hydrobromic acid should be administered frequently, and continued until the petechial spots have disappeared from the skin, in doses, of course, commensurate only with the hyperpyrexia or excess of heat.

Some prefer a solution of quinine in hydrobromic acid which may be administered in doses of from ½ to 1 drachm of the acid and 1 to 2 grs. of quinine hourly. The surface of the body should be regularly sponged as in other fevers. It is claimed that this mode of treatment will save over 75 per cent. of such cases, and prevent the distressing sequelæ which sometimes follow, shewing defective nerve power. The leeching is indispensable to relieve the violent head symptoms at the outset, and the anti-pyretic properties of the quinine are needful; but without the acid neither of these remedies would prove of more than temporary benefit.

The use of calomel has been much lauded by some, but is rapidly falling into disuse as unnecessary. Opium in moderate doses is of great service in the later stages of the disease and assists materially in promoting convelescence.—*Canada Lancet.*

