# Hydrobromic Acid—Correction

**Published:** 1878-06-20

**Authors:** 


### HYDROBROMIC ACID—CORRECTION.

By some unaccountable oversight, an article on *Hydrobromic Acid* occurred twice in our May number. This, of itself, is not a matter of much importance, and was excused, we trust, by the good sense of our readers, but the oversight is aggravated by the use of the *drachm* mark in a formula in the one and the *ounce* mark in the other. The latter is correct. We notice the same discrepancy in different journals into which the article has been copied. The oz. mark should be used in the preparation of the acid as it appears on page 147.

De Witt C. Wade, of Michigan, prepares the hydrobromic acid as follows :

℞.—Bromide Potass....................oz. xi.
Crystal. Tartaric Acid................oz. xiv.
Water..............................oz. xl.

Dissolve the bromide in the water; then the tartaric acid, and keep at a low temperature until precipitation ceases.

